# Handgrip strength is positively related to blood pressure and hypertension risk: results from the National Health and nutrition examination survey

**DOI:** 10.1186/s12944-018-0734-4

**Published:** 2018-04-17

**Authors:** Chao Ji, Liqiang Zheng, Rui Zhang, Qijun Wu, Yuhong Zhao

**Affiliations:** 1 Department of Clinical Epidemiology, Shengjing Hospital of China Medical University, No. 36, San Hao Street, Shenyang, 110004 Liaoning China; 2 Institute of Chronic Disease, Liaoning Provincial Center for Disease Control and Prevention, Shenyang, China

**Keywords:** Systolic blood pressure, Diastolic blood pressure, Handgrip strength, Hypertension, Body mass index

## Abstract

**Background:**

Isometric handgrip resistance exercise, a nonpharmacological lifestyle modification, has been recommended as a first-line treatment for hypertension. This study aimed to examine the relationship of handgrip strength to blood pressure and the risk of hypertension.

**Methods:**

The responses and examination of 4597 participants in the National Health and Nutrition Examination Survey (NHANES) were analyzed in this study. Systolic blood pressure (SBP) and diastolic blood pressure (DBP) were transformed to age- and sex-specific z-scores. Handgrip strength was adjusted by weight (kg) and converted to an age- and sex-specific z-score. The relationships of SBP and DBP to handgrip strength were analyzed by Pearson correlation test and multivariable linear regression. Binary logistic regression was used to analyze the association between handgrip strength and prevalence of hypertension.

**Results:**

Handgrip strength was positively related to higher DBP in men and women. In men, logistic regression models revealed that increased handgrip strength was associated with higher risk of hypertension after adjusting for age, BMI, smoking and drinking status; OR was 1.24 (95%CI: 1.04–1.48). After stratifying on BMI, handgrip strength was significantly associated with higher risk of hypertensions after adjusting for age, BMI, smoking and drinking status in overweight and obese men; OR was 1.31 (95%CI: 1.05–1.63). No significant associations were observed in women.

**Conclusions:**

Increased handgrip strength is associated with higher DBP in men and women. In men, especially overweight and obese men, strong handgrip strength may be associated with higher risk of hypertension.

**Electronic supplementary material:**

The online version of this article (10.1186/s12944-018-0734-4) contains supplementary material, which is available to authorized users.

## Background

Hypertension, also known as high or raised blood pressure (BP), is a condition in which the pressure in the arteries is persistently elevated. It contributes to the burden of heart disease, stroke, kidney failure, premature mortality and disability. Cardiovascular disease accounts for approximately 17 million deaths a year, which is nearly 1/3 of annual global deaths [1]. Every year, worldwide, 9.4 million people deaths die from complications of hypertension, which has become a global public health problem [2]. Risk factors increased the prevalence of hypertension include population growth, increased age and behavioral risk factors, such as unhealthy diet, tobacco use, consumption of alcohol, excess weight, exposure to persistent stress, high cholesterol, diabetes mellitus, and lack of physical activity. Strategies that have been implemented to prevent and manage hypertension include reducing exposure to behavioral risk factors and early detection and treatment of hypertension [3]. Despite these strategies, the mortality trend for hypertension continues to increase [4].

Current national and international guidelines for prevention of hypertension recommend that adults perform muscle strengthening activities 2 or more days a week to obtain at least 150 min/week of moderate-intensity or perform 75 min/week of vigorous-intensity aerobic physical activity [5–8]. Evidence indicates that isometric resistance training is an effective exercise modality to lower resting BP in both normotensive and hypertensive populations [9–12]. The handgrip strength test, a quick and inexpensive way to measure an individual’s muscle strength, is verified to be a satisfactory indicator of resistance training in an epidemiological study [13–15].

Although most evidence indicates that greater handgrip strength is associated with lower BP [16–19], some of them are limited to small sample size studies [19]. And other studies did not find this association [20, 21]. In contrast, in the oldest old persons, higher BP was found to be associated with greater handgrip strength [22]. Dong [14] and Diez-Fernandez [23] suggested that the effect of strong handgrip strength was mediated by body mass index (BMI). However, after adjustment for BMI, strong handgrip strength was still associated with higher, not lower, adolescent BP [14]. Analysis of the association between handgrip strength and BP adjusted for BMI in a large-scale population is relatively scarce.

Therefore, using a representative sample from the database of the National Health and Nutrition Examination Survey (NHANES) 2011–2012 and 2013–2014 survey participants aged ≥18 years old, we examined the association between handgrip strength and BP in adults after stratifying by, or adjusting for, age, sex and BMI. This study may have important implications for hypertension intervention measures.

## Methods

### Participants and samples

The NHANES is a cross-sectional study that examines the health and nutrition of children and adults in the United States. It collects data about demographics, health and health behaviors. NHANES uses a complex stratified, multistage, probability cluster sample designed to represent the U.S. population with respect to age, sex and race [24, 25].

Handgrip dynamometer assessment was recently included in NHANES. Handgrip strength data is available in the 2011–2012 and 2013–2014 survey cycles. Handgrip strength data from the participants aged ≥18 years old who participated in these survey cycles were analyzed in this study. Written informed consent was obtained from all the participants. The NHANES protocol was reviewed and approved by the National Center for Health Statistics Research Ethics Review Board.

### Anthropometry

Height was measured to the nearest 0.1 cm using a digital stadiometer connected to an acrylic headpiece that interfaced directly with the Integrated Survey Information System (ISIS) anthropometry software application. Weight was measured in kilograms used a digital weight scale. BMI was calculated as weight in kilograms divided by height in meters squared (kg/m^2^), which was categorized as underweight, normal, overweight and obese according the NIH criteria [26].

### Blood pressure

BP measurements were taken during the NHANES one-examination visit. All participants sat all the way to the back of the chair to keep the spine straight and rested quietly for 5 min prior to BP measurement. The arm was bare and unrestricted by clothing with the palm of the hand turned upward. The elbow slightly flexed and positioned so that the midpoint of the upper arm was at the level of the heart. Three consecutive BP readings were obtained by the auscultatory or oscillatory method. If a BP measurement was interrupted or incomplete, a fourth measurement was performed. All BP determinations (systolic and diastolic) were performed in the NHANES mobile examination center. Hypertension was defined as resting BP persistently at or above 140/90 mmHg according to the international standard [27].

### Handgrip strength

Handgrip strength, as an indicator of muscle strength, was evaluated using a handgrip strength test according to the recommendations of the Institute of Medicine [28]. Takei Digital Grip Strength Dynamometer, Model T.K.K.5401 (Takei Scientific Instruments Co., Niigata, Japan) was used for all muscle strength assessments. Each hand was tested three times, alternating hands with a 60-s rest between measurements on the same hand. Before measurement of handgrip strength, the dynamometer was adjusted to participants’ hands size, until the second joint of the index finger was at a 90-degree angle to the handle. The placements of the handle were marked for each hand at the same time. Participants stood straight with the feet hip with apart and arms fully extended along side of thigh with palms facing the thigh. The hand, which was in line with the wrist and forearm, performed a quick, maximally hard squeeze of the handle. The complete handgrip strength testing protocol is described in NHANES muscle strength procedures manual.

### Statistical analysis

Descriptive characteristics were expressed as mean ± standard deviation (SD) for continuous variables and as frequency with percentage for categorical variables. Baseline characteristics between men and women were compared using t test and chi-square test.

To avoid the potential bias effect of body weight on the estimation of handgrip strength, relative handgrip strength [handgrip (kg)/body weight (kg)] was calculated to assess the handgrip strength. This adjustment was based on standard assumptions about morphological effects [13]. Because distribution of handgrip strength, SBP and DBP varied by age and sex, these parameters were transformed into sex and age specific SD scores (z-scores) in the analysis of the association between handgrip strength and risk of hypertension by binary logistic regression (z-score = (value − mean)/SD) [29]. Pearson correlation test, partial correlation and linear regression analysis were used to analyze the relationships between handgrip strength, BMI, SBP and DBP. Binary logistic regression was used to analyze the association between handgrip strength and prevalence of hypertension.

## Results

Baseline characteristics of the study participants are shown in Table 1. Our study included 4597 participants; mean age was 47.4 ± 18.3 years. Compared with women, men had higher height, weight, BMI, handgrip strength, SBP and DBP. However, the gender-related difference in hypertension prevalence was not significant.

**Table 1:** Baseline characteristics of study subjects by gender

	All (4597)	Men (2184)	Women (2413)	*t/χ* ^*2*^	*P*
Age (yrs)	47.4 ± 18.3	47.1 ± 18.3	47.8 ± 18.3	1.322	0.186
Height (cm)	167.1 ± 10.1	174.3 ± 7.8	160.5 ± 7.1	61.562^a^	< 0.01
Weight (kg)	81.5 ± 22.4	86.9 ± 21.8	76.5 ± 21.7	15.776^a^	< 0.01
BMI (kg/m^− 2^)	29.1 ± 7.3	28.5 ± 6.5	29.6 ± 7.9	5.360^a^	< 0.01
*BMI Category*					
Underweight	84 (1.9)	38 (1.8)	46 (2.0)	63.541^b^	< 0.01
Normal	1278 (29.0)	594 (28.3)	684 (29.6)		
Overweight	1368 (31.1)	767 (36.6)	601 (26.1)		
Obese	1674 (38.0)	698 (33.3)	976 (42.3)		
Handgrip strength (kg)	33.9 ± 10.4	41.5 ± 8.8	26.7 ± 5.7	61.850^a^	< 0.01
SBP (mm Hg)	122.8 ± 17.7	124.5 ± 16.3	121.2 ± 18.8	6.197^a^	< 0.01
DBP (mm Hg)	69.7 ± 11.4	70.8 ± 12.0	68.8 ± 10.8	5.562^a^	< 0.01
Smoking	1944 (42.4)	1096 (50.4)	848 (35.2)	107.976^b^	< 0.01
Drinking	2855 (70.0)	1579 (80.5)	1276 (60.2)	199.285^b^	< 0.01
Hypertension	715 (16.7)	355 (17.3)	360 (16.1)	0.985	0.321

Data are presented as mean ± SD or count (%)

^a^Difference between men and women was significant using *t*- test

^b^Difference between men and women was significant using chi-square test

*BMI* body mass index, *SBP* systolic blood pressure, *DBP* diastolic blood pressure

The relationships of handgrip strength with BMI, SBP and DBP were analyzed using a separate Pearson correlation test for each pair of variables. In all participants, higher handgrip strength was correlated with lower SBP (*r* = − 0.084, *P* < 0.01), DBP (*r* = − 0.034, *P* < 0.05) and BMI (*r* = − 0.691, *P* < 0.01). BMI was positively correlated with SBP and DBP (both, P < 0.01). The similar results were observed in women. In men, higher BMI was also correlated to higher SBP and DBP (both, P < 0.01); however, handgrip strength SBP and DBP were not significantly correlated with each other.

While controlling for BMI in partial correlation tests, handgrip strength increased with SBP (*r* = 0.033, *P* < 0.05) and DBP (*r* = 0.094, *P* < 0.01) in all subjects. The similar correlations were observed in men. However, in women handgrip strength was only positively correlated to DBP.

The associations between BP and handgrip strength, based on linear regression models, are shown in Additional file 1: Table S1 and Table S2. SBP was positively associated with handgrip strength (1 SD higher increase in handgrip strength was related to 0.065 SD higher SBP, *P* < 0.01) in men after adjusting for age and BMI (model 2). After also adjusting for smoking and drinking status (model 3), the regression coefficient changed minimally; however, the association was no longer significant (*P* = 0.062). Significant association was also observed between SBP and unadjusted handgrip strength in women (model 1). However, in women, no significant associations were founded between handgrip strength and SBP in adjusted models 2 and 3. Higher handgrip strength was significantly associated with higher DBP in men: One SD increase in handgrip strength was related to 0.165 SD higher in DBP in men after adjusting for age and BMI, *P* < 0.01 (model 2). In men, after also adjusting for smoking and drinking, one SD increase in handgrip strength was related to 0.155 SD higher DBP (model 3). In contrast to the lack of association between handgrip strength and SBP in women, DBP was significantly association with handgrip strength in women. Unadjusted handgrip strength was negatively associated with DBP (model 1). After adjusting for age and BMI, one SD increase in handgrip strength in women was related to 0.096 SD higher DBP (model 2) and 0.081 SD higher in DBP after also adjusting for smoking and drinking status (model 3).

Table 2 shows the association between handgrip strength and hypertension, stratified by gender; binary logistic regression was used to estimate the odds ratio (OR) of handgrip strength for the risk of hypertension. Unadjusted handgrip strength was not associated with risk of hypertension in men (model 1). After adjusting for age and BMI (model 2) and smoking and drinking status addition (model 3), significant associations were observed: ORs were 1.229 (95%CI: 1.036–1.458) and 1.241 (95%CI: 1.038–1.484), respectively. No significant associations between handgrip strength and hypertension were observed in women.

**Table 2:** Association between handgrip strength and hypertension stratified by gender

	Men (*N* = 1587)				Women (*N* = 1960)			
	*β*	S.E.	OR (95% CI)	*P*	*β*	S.E.	OR (95% CI)	*P*
*Model*								
Model 1: unadjusted	− 0.083	0.058	0.921 (0.822, 1.032)	0.154	−0.098	0.071	0.907 (0.789, 1.043)	0.169
Model 2: adjusted for age and BMI	0.206	0.087	1.229 (1.036, 1.458)	0.018	0.007	0.117	1.007 (0.800, 1.268)	0.950
Model 3: as Model 2 and smoking and drinking	0.216	0.091	1.241 (1.038, 1.484)	0.018	−0.009	0.122	0.991 (0.781, 1.257)	0.939

*BMI* body mass index. Handgrip was transformed into age and sex specific SD scores (z-score)

Although participants were first categorized by the international classification of adult underweight, normal, overweight, and obesity according to BMI value, to perform BMI-stratified analysis of the association between handgrip strength and hypertension these four weight categories were combined into Underweight & Normal (BMI < 25) and Overweight & Obese (BMI ≥25) groups to categorize all participants into two BMI strata. Table 3 shows the association in men between handgrip strength and hypertension risk stratified by these weight groups. No significant associations were observed in the normal/underweight group. In the overweight/obese group, unadjusted handgrip strength was not associated with hypertension risk (model 1). After adjusting for age and BMI, the association between handgrip strength and hypertension was significant (*P* < 0.05, model 2); the OR was 1.288 (95%CI: 1.041, 1.594). After adjusting also for smoking and alcohol status, this association did not changed (*P* < 0.05, model 3); the OR was 1.311 (95%CI: 1.053, 1.631). In women, similar to the results in Table 2, no significant associations were found between handgrip strength and hypertension risk (Table 4).

**Table 3:** Association in men between handgrip strength and hypertension stratified by two BMI categories

	Underweight & Normal (*N* = 563)				Overweight & Obese (*N* = 1292)			
	*β*	S.E.	OR (95%CI)	*P*	*β*	S.E.	OR (95%CI)	*P*
*Model*								
Model 1: unadjusted	−0.106	0.127	0.899 (0.701, 1.153)	0.402	−0.047	0.079	0.954 (0.817, 1.115)	0.557
Model 2: adjusted for age and BMI	0.127	0.158	1.136 (0.833, 1.549)	0.421	0.253	0.109	1.288 (1.041, 1.594)	< 0.05^*^
Model 3: as Model 2 and smoking and drinking	0.030	0.171	1.030 (0.737, 1.440)	0.863	0.270	0.112	1.311 (1.053, 1.631)	< 0.05^*^

*BMI* body mass index. All participants were categorized into two strata according to BMI value: Underweight & Normal (BMI < 25) and Overweight & Obese (BMI ≥25). Handgrip was transformed into age and sex specific SD scores (z-score). * *P* < 0.05

**Table 4:** Association in women between handgrip strength and hypertension stratified by two BMI categories

	Underweight & Normal (*N* = 636)				Overweight & Obese (*N* = 1323)			
	*β*	S.E.	OR (95%CI)	*P*	*β*	S.E.	OR (95%CI)	*P*
*Model*								
Model 1: unadjusted	−0.163	0.154	0.849 (0.628, 1.150)	0.291	−0.043	0.104	0.958 (0.782, 1.174)	0.679
Model 2: adjusted for age and BMI	−0.133	0.211	0.876 (0.579, 1.326)	0.531	0.022	0.152	1.022 (0.759, 1.377)	0.885
Model 3: as Model 2 and smoking and drinking	−0.123	0.215	0.884 (0.580, 1.348)	0.568	0.008	0.158	1.008 (0.740, 1.373)	0.961

*BMI* body mass index. All participants were categorized into two strata according to BMI value: Underweight & Normal (BMI < 25) and Overweight & Obese (BMI ≥25). Handgrip was transformed into age and sex specific SD scores (z-score)

## Discussion

The present study revealed that handgrip strength was positively and significantly associated with DBP in men and women. There were no significant associations between handgrip strength and SBP in women. Handgrip strength appears to increase the risk of hypertension in men especially for those who were overweight and obese (BMI ≥ 25). However, the same results were not observed in women.

Emerging evidence has demonstrated the effect of isometric handgrip exercise on reducing BP in normotensive and hypertensive populations [30]. Meta-analyses of randomized controlled trials revealed that isometric handgrip training could reduce resting BP [19, 31]. Recent randomized trials also support the same conclusion [16, 17]. Possible mechanisms for these effects include improvements in conduit and resistance due to vessel endothelium dependent dilation, oxidative stress and autonomic regulation of heart rate and BP [30]. However, most of these studies included relatively few subjects, and the exercise trainings were not uniform. Recent studies report variable findings about the association between handgrip strength and BP. A recent randomized controlled trial found that isometric handgrip exercise did not decrease BP [20]. In 2010, Dong et al. [14] conduct a survey of 89,655 adolescents in China aged 13–17 years; their results indicated that strong handgrip strength was associated with increased adolescent BP. Another longitudinal cohort study conducted by Taekema et al. reported that higher BP is associated with greater handgrip strength in the oldest old, aged over 85 years [22]. In their middle-age subjects, BP and handgrip strength were not significantly associated.

The association between BP and handgrip strength in adolescents may be confounded by BMI. Few of these studies adjusted for BMI; such adjustment was suggested, particularly in samples of younger adults [23, 32–34]. The present study revealed that handgrip strength was positively related to BP, the inverse of this relationship before adjusting for BMI. Moreover, that association did not change after controlling for smoking and drinking status. Similar results were also seen in the association between handgrip strength and risk of hypertension. These findings indicated that handgrip strength was positively associated with BP and the risk of hypertension.

Several mechanisms could explain the positive associations between handgrip strength and BP. Peripheral vascular resistance increases with chronological age due to reduced sympatholysis, which results in an elevated sympathetic tone [35, 36], or vascular resistance increased with morphological changes in the arteriolar network [37]. Another possible explanation is that BP is associated with the age-associated loss of lean mass [38, 39]. In addition, handgrip strength is probably associated with other risk factors for hypertensions, such as metabolic syndrome or cardiovascular disease biomarkers, including triglyceride, HDL, LDL, glycohemoglobin (HbA1c), uric acid, insulin resistance and serum adiponectin levels [40–44]. Although the exact mechanisms of handgrip strength induced lipids changes and interaction with hypertension remain unclear, several potential hypotheses have been proposed. Adiponectin directly stimulates the activation of muscle autophagy, which could affect the type and size of muscle cells [41]. The association between handgrip strength and metabolic syndrome seemed to be mediated by increasing insulin resistance [42]. Insulin resistance stimulates muscle glucose uptake and increases body fat. A higher percentage of body fat generally translates to a higher rate of appearance of free fatty acids in plasma, which could also induce higher blood pressure [44]. Thus, considering the numerous factors associated with BP and handgrip strength, the mechanism of the relationship between BP and handgrip strip strength has not yet been fully explained; clarification requires further study.

This study had several strengths for studying the association between handgrip strength and BP and risk of hypertension. The National Health and Nutrition Examination Survey (NHANES) is a program of studies designed to assess the health and nutritional status of adults and children in the United States, which examines a nationally representative sample and can be generalized to western populations. Secondly, in this study, handgrip strength was adjusted for age, BMI, and smoking and drinking status. Few studies of handgrip strength are adjusted for body size and composition, although that was suggested in previous studies [45]. A weakness of this study was that simply finding an association between handgrip strength and BP and risk of hypertension does not support a causal relationship because this study used a cross-sectional design. Longitudinal data and clinical trials are needed to clarify whether the relationship between muscle strength and BP and risk of hypertension is causal. Another limitation is that findings based on the NHANES database may not be generalizable to non-western populations; this study needs to be replicated in other populations to better understand the external validity of our findings.

## Conclusion

The current study shows that handgrip strength is significantly associated with lower BP. In contrast to most previous reports, this study found that strong handgrip strength was related to increased DBP, and greater handgrip strength was associated with greater the risk of hypertension after adjusting for age, BMI, smoking and drinking status, which were closely related to BP. Further clinical trials and cohort studies from large scale samples are needed to verify these results and explain the possible mechanisms of this relationship. This study may have important relevance for hypertension intervention.

## Additional file

Additional file 1: **Table S1.** Association between handgrip strength and SBP adjusted for age and BMI stratified by gender. **Table S2.** Association between handgrip strength and DBP stratified by gender. (DOCX 15 kb)

## Abbreviations

BMI: Body mass index
BP: Blood pressure
DBP: Diastolic blood pressure
NHANES: National Health and Nutrition Examination Survey
SBP: Systolic blood pressure
SD: Standard deviation
