# Supplementary material for: Handgrip strength is positively related to blood pressure and hypertension risk: results from the National Health and nutrition examination survey
Source: Lipids Health Dis. 2018 Apr 17;17:86. doi: 10.1186/s12944-018-0734-4 (PMC5904981; doi:10.1186/s12944-018-0734-4)
Supplement: Supplementary file 1 — Table S1. Association between handgrip strength and SBP adjusted for age and BMI stratified by gender. Table S2. Association between handgrip strength and DBP stratified by gender. (DOCX 15 kb) [file 12944_2018_734_MOESM1_ESM.docx]

Additional file

**Supplementary:**

**Table S1.** Association between handgrip strength and SBP adjusted for age and BMI stratified by gender

|  | Men (*N*=1,859) | | | |  | Women (*N* =1,963) | | | |
| --- | --- | --- | --- | --- | --- | --- | --- | --- | --- |
|  | *β* (95%CI) | S.E. | Standard -ized *β* | *P* |  | *β*(95%CI) | S.E. | Standard -ized *β* | *P* |
| *Model* |  |  |  |  |  |  |  |  |  |
| Model 1: unadjusted | -0.038 (-0.077, 0.002) | 0.020 | -0.043 | 0.062 |  | -0.142 (-0.190, -0.094) | 0.025 | -0.129 | <0.01^**^ |
| Model 2: adjusted for age and BMI | 0.057 (0.004, 0.110) | 0.027 | 0.065 | 0.036 |  | -0.010 (-0.080, 0.060) | 0.036 | -0.009 | 0.778 |
| Model 3: as Model 2 and smoking and drinking | 0.054 (-0.001, 0.109) | 0.028 | 0.062 | 0.054 |  | -0.018 (-0.090, 0.055) | 0.037 | -0.016 | 0.632 |

BMI, body mass index. SBP, systolic blood pressure. Handgrip and SBP were transformed into age and sex specific SD scores(z-score). ** P<0.01

**Table S2.** Association between handgrip strength and DBP stratified by gender

|  | Men (*N* =1855) | | | |  | Women (*N* =1957) | | | |
| --- | --- | --- | --- | --- | --- | --- | --- | --- | --- |
|  | *β*(95%CI) | S.E. | Standard -ized *β* | *P* |  | *β* (95%CI) | S.E. | Standard -ized *β* | *P* |
| *Model* |  |  |  |  |  |  |  |  |  |
| Model 1: unadjusted | -0.006 (-0.049, 0.038) | 0.022 | -0.006 | 0.799 |  | -0.072 (-0.118, -0.026) | 0.023 | -0.069 | <0.01^**^ |
| Model 2: adjusted for age and BMI | 0.157 (0.099, 0.215) | 0.030 | 0.165 | <0.01 |  | 0.099 (0.032, 0.167) | 0.034 | 0.096 | <0.01^**^ |
| Model 3: as Model 2 and smoking and drinking | 0.146 (0.086, 0.206) | 0.031 | 0.155 | <0.01 |  | 0.085 (0.015, 0.155) | 0.035 | 0.081 | <0.05^*^ |

BMI, body mass index; DBP, diastolic blood pressure. Handgrip and DBP were transformed into age and sex specific SD scores (z-score). * P<0.05; ** P<0.01
